# Deep Mouse Brain Two-Photon Near-Infrared Fluorescence Imaging Using a Superconducting Nanowire Single-Photon Detector Array

**DOI:** 10.1021/acsphotonics.4c00111

**Published:** 2024-09-11

**Authors:** Amr Tamimi, Martin Caldarola, Sebastian Hambura, Juan C. Boffi, Niels Noordzij, Johannes W. N. Los, Antonio Guardiani, Hugo Kooiman, Ling Wang, Christian Kieser, Florian Braun, Mario A. Usuga Castaneda, Andreas Fognini, Robert Prevedel

**Affiliations:** † Cell Biology and Biophysics Unit, European Molecular Biology Laboratory, Heidelberg 69117, Germany; ‡ Single Quantum B.V, Delft, HH 2629, The Netherlands; § Chemical Synthesis Core Facility, European Molecular Biology Laboratory, Heidelberg 69117, Germany; ∥ Developmental Biology Unit, European Molecular Biology Laboratory, Heidelberg 69117, Germany; ⊥ Epigenetics and Neurobiology Unit, European Molecular Biology Laboratory Rome, Monterotondo 00015, Italy; # German Center for Lung Research (DZL), Heidelberg 69120, Germany; ∇ Interdisciplinary Center of Neurosciences, Heidelberg University, Heidelberg 69120, Germany

**Keywords:** two-photon microscopy, deep brain imaging, short-wave infrared region, NIR dyes, superconducting nanowire single-photon detector

## Abstract

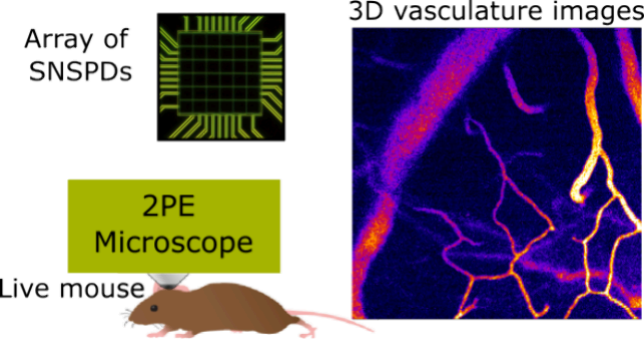

Two-photon microscopy (2PM) has become an important tool in biology to study the structure and function of intact tissues *in vivo*. However, adult mammalian tissues such as the mouse brain are highly scattering, thereby putting fundamental limits on the achievable imaging depth, which typically reside at around 600–800 μm. In principle, shifting both the excitation as well as (fluorescence) emission light to the shortwave near-infrared (SWIR, 1000–1700 nm) region promises substantially deeper imaging in 2PM, yet this shift has proven challenging in the past due to the limited availability of detectors and probes in this wavelength region. To overcome these limitations and fully capitalize on the SWIR region, in this work, we introduce a novel array of superconducting nanowire single-photon detectors (SNSPDs) and associated custom detection electronics for use in near-infrared 2PM. The SNSPD array exhibits high efficiency and dynamic range as well as low dark-count rates over a wide wavelength range. Additionally, the electronics and software permit a seamless integration into typical 2PM systems. Together with an organic fluorescent dye emitting at 1105 nm, we report imaging depth of >1.1 mm in the in vivo mouse brain, limited mostly by available labeling density and laser properties. Our work establishes a promising, and ultimately scalable, new detector technology for SWIR 2PM that facilitates deep tissue biological imaging.

## Introduction

Light microscopy provides a noninvasive and high-resolution, optical way to study biological structure and function. However, in many mammalian tissues, light attenuation, i.e., scattering and absorption, poses a grand challenge that often prevents investigations of cells and processes seated inside deep, yet physiologically relevant, tissues. Over the past decades, multiphoton excitation and in particular, two-photon excitation microscopy (2PM), has become the gold standard for recording cellular structure and function inside scattering tissues such as the mouse brain in vivo.^1,2^ However, the maximum penetration depth of two-photon microscopes is fundamentally limited by the onset of out-of-focus fluorescence near the surface with increasing excitation power and typically reaches 600–800 μm depth inside the mammalian brain.^3^ Alternatively, higher-order fluorescence excitation, notably three-photon excitation microscopy (3PM), has shown potential for deeper imaging beyond 1 mm,^4−8^ yet the significantly lower 3P cross section compared to 2P demand diligent optimization of excitation laser sources and associated parameters in order to prevent potential photodamage.^5,9,10^ Shifting the excitation wavelength further into the SWIR reduces tissue scattering and thus affords higher tissue penetration depth, as established by pioneering^11^ as well as recent work^12,13^ that achieved impressive overall image depth. For example, both refs (12 and 13) utilized custom-built, energetic femtosecond lasers operating at 1700 nm together with highly engineered fluorescence probes to visualize blood vessels up to 1.8 and 2.2 mm imaging depth in the live mouse brain, respectively. This established the potential of the red-shifted SWIR wavelength region for deep imaging, yet prior work was limited by the use of inefficient SWIR detectors, as current InGaAS-based photomultiplier tubes (PMTs) have fairly poor quantum efficiency (Q.E. < 2.5%) and high dark counts (∼10^5^ s^–1^) compared to visible PMTs.

Recently, high-gain, low-noise, and high-efficiency detectors based on superconducting nanowires single photon detectors (SNSPD) have been developed, which show unprecedented performance in the SWIR region.^14,15^ These properties make them eminently suitable for many applications, including near-infrared microscopy. Previous work has successfully demonstrated their use for confocal bioimaging applications using quantum dots.^16,17^ However, the relatively bright quantum dot fluorescence relied on one-photon excitation by a continuous-wave NIR laser and hence did not provide the intrinsic optical sectioning and scattering resilience afforded by 2P excitation. Furthermore, only ballistic fluorescence was detected through a pinhole, therefore wasting precious signals. For efficient 2P-excitation in deep tissues, low-repetition rate, fs-pulsed lasers have proven to be optimal.^3,10^ With such excitation sources, 2/3PM typically operates in the single laser pulse per pixel regime, which in turn necessitates pixelated, large area detectors to achieve a sufficiently high dynamic range to visualize different levels of fluorescence emanating from each individual voxel. As SNSPDs intrinsically operate in the single-photon detection regime and their sensing area is typically very small (∼100 μm^2^), here we developed a novel SNSPD array composed of 36 (6 × 6) individual detectors of ∼3600 μm^2^ total area. This layout substantially enhances both the dynamic range thanks to the pixelation but also the effective detector area, which improves overall light collection efficiency as nonballistic fluorescence photons can also be captured.

**Figure 1:**
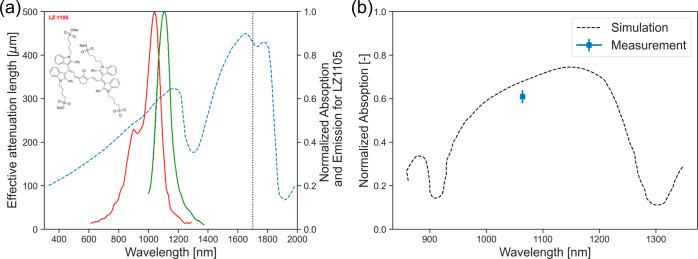
Spectral characteristics of NIR dye and SNSPD array. (a) The blue dashed line depicts the effective attenuation length (left axis) for brain tissue and highlights the improved penetration in the SWIR regime (1000–1700 nm). Data extracted from ref (19). The vertical dotted line depicts the laser source used for two-photon excitation, while the solid curves depict the (normalized) absorption and emission spectra of the LZ1105 dye (right axis). LZ1105 spectra taken from ref (18). (b) Spectral characterization of the SNSPD array. The dashed curve is a numerical simulation of the expected spectral response of the array of SNSPDs while the square represents the measured array efficiency at 1064 nm. Note that the simulation is usually an upper bound for the efficiency, since it does not incorporate material imperfections.

To demonstrate the potential of these novel class of SWIR detectors in proof-of-principle experiments, we synthesized a near-IR organic dye^18^ that can be 2P excited at 1700 nm and whose emission at ∼1100 nm overlaps with the sensitivity and Q.E. peak of the SNSPD array (Figure 1a,b). Together with the SNSPD array and custom read-out electronics, we demonstrate deep tissue in vivo microscopy of the adult mouse brain vasculature down to approximately 1.1 mm depth, which matches physically achievable 2P depths^3^ and surpasses routine 2PM depths by a few hundred micrometers. Our work establishes SNSPD arrays as versatile, easy-to-use detectors for 2PM and clearly demonstrates the potential of this technology for deep tissue imaging, paving the way for further developments of the technology as well as long-wavelength fluorophores and excitation sources.

## Results

### Development, Characterization, and Integration of the SNSPD Array

Superconducting nanowire single-photon detectors (SNSPDs) are a pioneering new light detection technology in the SWIR region and have so far found numerous applications in quantum communication and quantum optics.^20^ They are based on superconducting nanowires and combine outstanding detection efficiency with very high time resolution and low dark counts^14^ that vastly surpass photomultipliers and APDs in the wavelengths of interest (1000–2500 nm) in terms of sensitivity and time resolution. For a full overview of detector technologies in both the visible as well as SWIR regimes, see SI Figure 3 and SI Table 1.

By far, the most common implementation of SNSPDs consists of a fiber-coupled system, where the light to be sensed is delivered to the SNSPD inside the cryostat by means of a single-mode optical fiber. For the specific case of 2P microscopy, such implementation would be disadvantageous since the single-mode fiber will limit dramatically the number of fluorescence photons to be collected as they contain a high fraction of scattered light. To overcome this, we developed a free-space SNSPD system in which the light reaches the detectors inside the cryostat through optical windows. Such a system benefits from the high efficiency and low dark noise of SNSPDs, which is instrumental in imaging faint fluorescence signals deep inside highly scattering tissues, such as the mouse brain.

**Figure 2:**
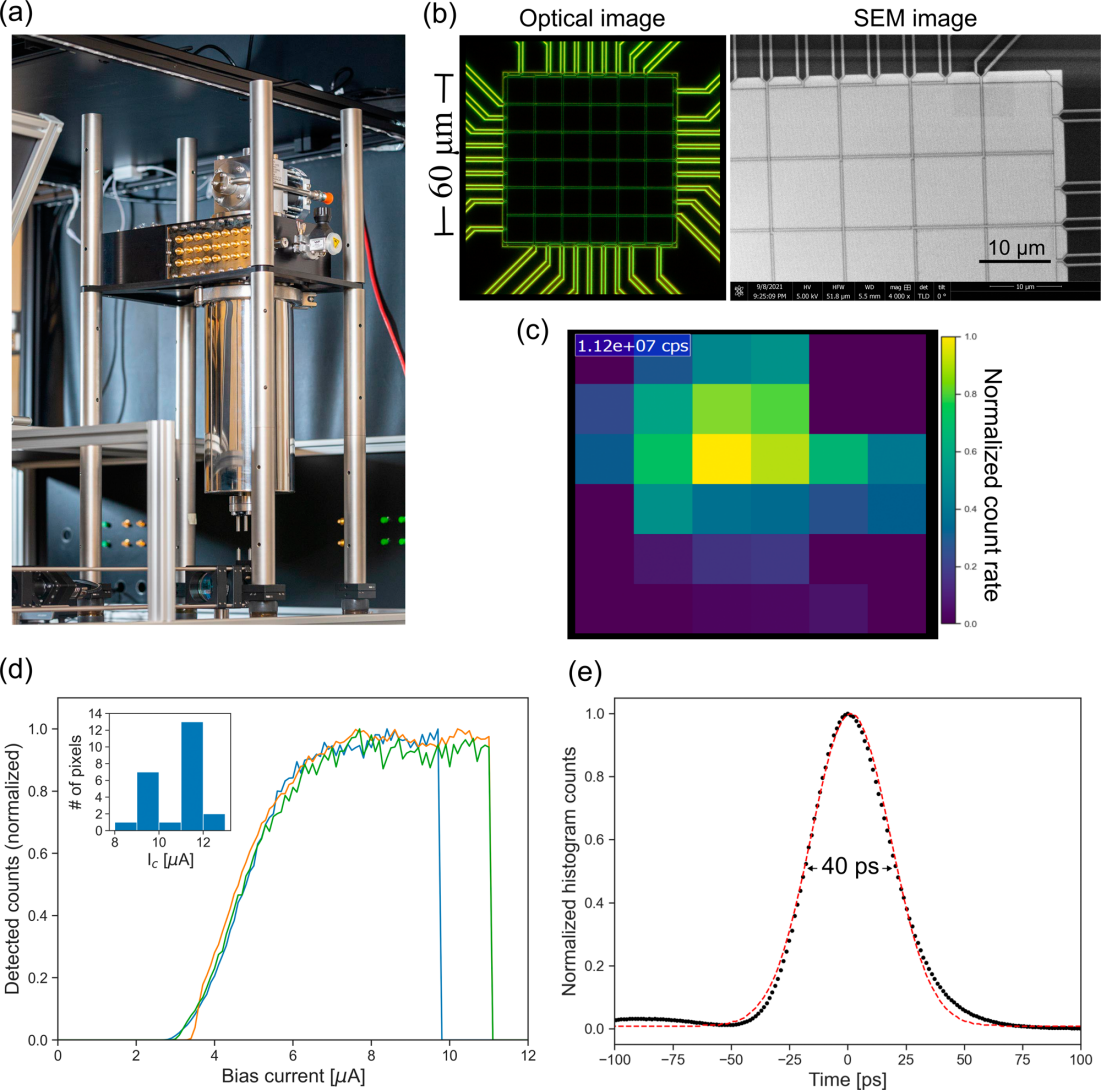
Schematics and characterization of the free-space coupled SNSPD array. (a) Picture of the free-space coupled cryostat on an optical table. (b) (left) Optical image (dark field mode) of the 6 × 6 array with a sensitive area of 60 μm × 60 μm, along with a scanning electron microscope (SEM) image of the top corner of the array (right). (c) Map of the detected fluorescence counts using the SNSPD array when incorporated in the 2PE microscope. The colormap shows normalized counts of the Gaussian profile while the inside number depicts the total number of counts detected by the complete array in 1 s. (d) Bias current sweep of three different pixels from the array, showing a good plateau indicating saturation of the internal efficiency. These curves also provide the critical current at which the SNSPD becomes nonsuperconducting and the counts go to zero. The inset shows a histogram of the measured critical currents (*I*_c_) for all the connected elements in the array. (e) Representative timing jitter histogram (black dots), with a fwhm of 40 ps (from the Gaussian fit, dashed red line; also see Methods).

The SNSPD system used in this work consists of a closed-cycle cryostat to cool the superconducting nanowires to ∼3K with the important addition of windows for free-space optical access. We fabricated an array detector that consists of 6 × 6 individual SNSPDs with square shape and 10 μm side length (including the readout lines), providing a total detection area of 60 μm × 60 μm. The sensitive area of the detector is an important parameter for two-photon excited microscopy as it increases the collection efficiency as a high fraction of the emitted photons typically experience scattering in the tissue and therefore will reach the detector plane in a different position with respect to the ballistic photons. Figure 2 shows a picture of the system on an optical table with the optical window facing down. The bias and readout of each pixel in the array is achieved through independent electrical lines, maintaining the capabilities for single-photon detection and the above-mentioned advantages of SNSPDs in the near-infrared range. However, to keep the size and costs of the readout electronics at bay, we only contacted 24 working pixels from the center of the 36 array; see Figure 2c. The rationale was that corner pixels will only contribute a minority to the overall signal.

A detailed characterization of our SNSPD array system and its main performance metrics is depicted in Figure 2c–e. Figure 2c depicts typical count rates of the array when 2PE fluorescence from LZ1105 is focused on the center pixel within a size of ∼15 μm, which ensures that all of the incident light is collected by the complete array. Figure 2d depicts a plot of the normalized detected counts for different bias currents for three representative pixels in the array. The measurements show a clear saturation (∼7–10 μA), indicating a good internal efficiency and critical currents (around 10 μA,) in which superconductivity and thus detection is lost.

We also characterized the SNSPD array quantum efficiency and found a total system efficiency of 57 ± 5% at a wavelength of 1064 nm (see the Methods section for more details). Since the measured transmission for the combined optical windows in the cryostat at 1064 nm is ∼0.935 and the transmission of the focusing lens is ∼0.99 at 1064 nm, we estimate that the SNSPD array itself has a slightly higher total detection efficiency of ∼61%, as shown in Figure 1b. We also characterized the so-called dark-count rate, i.e., the number of detection events when no input light is present, and obtained a total of 9.7 × 10^3^ s^–1^ for the complete array, which corresponds to <300 s^–1^ dark-count rate per pixel on average. This was achieved with no additional filtering or short pass filters, and spectral filtering of long wavelengths would likely lead to a further reduction in the dark-count rate. Note that a standard photomultiplier tube for the same wavelength range provides a typical quantum efficiency of 2% and comparable dark counts; therefore, we expect the SNSPD to yield 2PM images with a substantially better signal-to-noise ratio.

**Figure 3:**
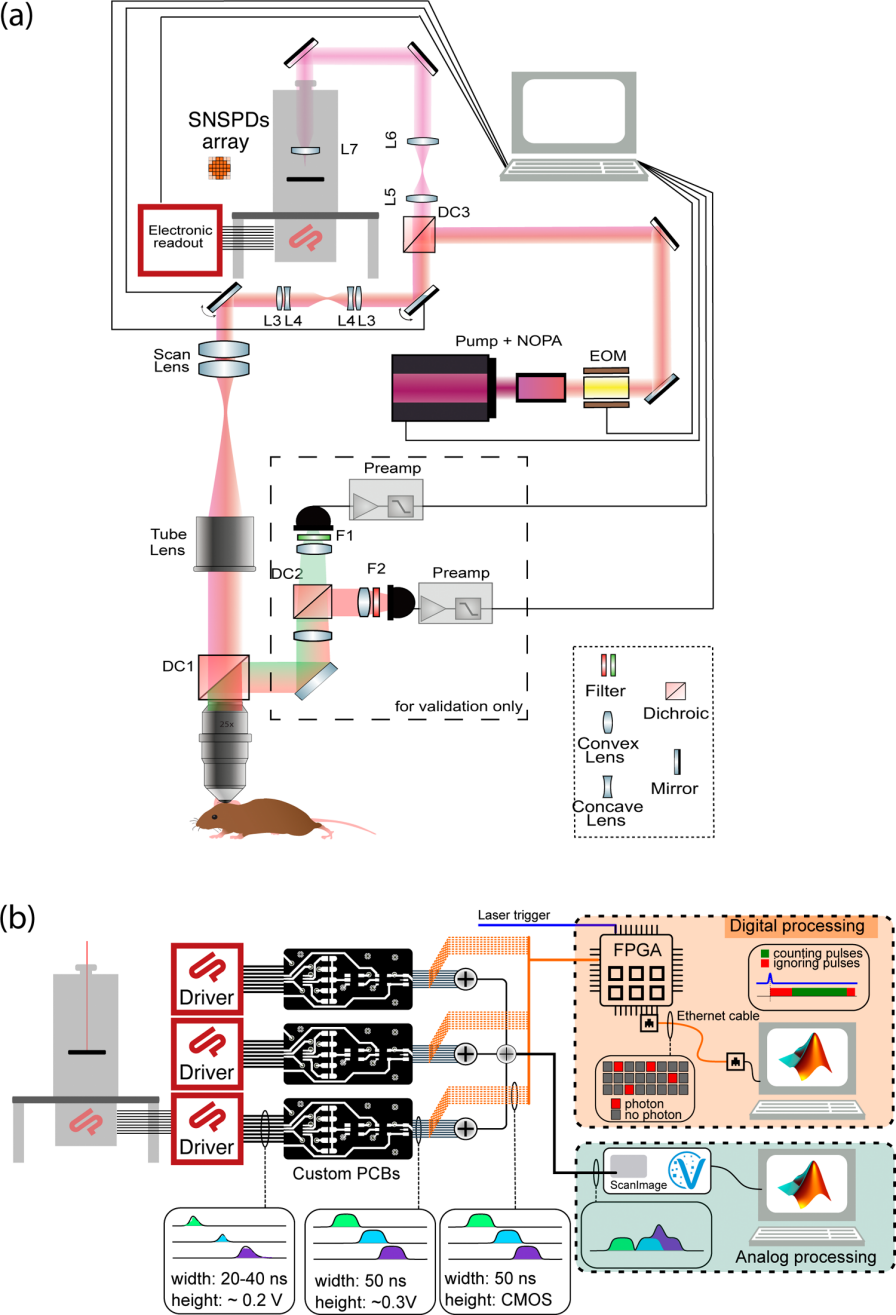
Integration of the SNSPD array into a custom MPM and dedicated signal processing pipeline. (a) Optical layout of the multiphoton microscope. The excited fluorescence is descanned and relayed onto the SNSPD array. Note that the dashed box containing traditional PMTs was only used during development but is not necessary during imaging. (b) Design of the SNSPD signal processing electronics. The 24 outputs of the SNSPD drivers are processed by custom PCBs and converted into TTL digital signals (3.3 or 5 V logic height) as well as analog signals. For the analogue processing, the signals are summed into one single analogue pulse that existing processing software and hardware can readily use. For the digital processing, a FPGA records the number of pulses (photons) for each channel separately, triggered by the excitation laser, and streamed to the computer for offline image reconstruction (also see Methods and SI Figure 2for details).

The SNSPD array was integrated into a custom multiphoton microscope optimized for deep tissue imaging^6,21,22^ (see Figure 3a and Methods). In contrast to typical multiphoton microscopy (MPM), however, the fluorescence signal is effectively descanned by the galvanometric mirrors before being separated from the excitation light using a dichroic mirror and then optically relayed into the cryostat and onto the SNSPD array.

### Electronic Interfacing and Integration

In order to have a functional SNSPD, a constant bias current must flow on the superconducting nanowire. Since the photon-detection event will break the superconductivity temporarily, the bias current is diverted to an amplification stage to generate output pulses of reasonable amplitude for further usage (a few 100 mV peak height), which is accomplished for 24 individual channels of the 6 × 6 array by dedicated electronics (Atlas, Single Quantum). This electronic driver provides an electrical analog output where the amplified pulses coming from the SNSPD can be sent to other electronics, such as a time-tagger or, as in our case, tailor-made electronics aimed to integrate the SNSPDs into an existing multiphoton microscope and their associated hardware control software.

In order to aid the seamless integration of the SNSPD array into common MPMs, we developed a custom processing electronics (Figure 3b and SI Figure 2), which provides dual functionality: First, the raw analogue SNSPD output pulses, which represent individual photon detection events, are converted to TTL pulses of fixed length and sent to an FPGA, which enables streaming the raw event outputs of all the channels to disk. Additional channels allow various trigger and synchronization signals to be saved simultaneously for offline image reconstruction. This maintains a high temporal (8 ns) resolution as well as full spatial resolution of the fluorescence signal. In parallel, the uniform digital pulses are further signal-conditioned with low-pass filters engineered to produce a smooth analog pulse without ringing (see Methods). The pulses from all 24 channels are summed to represent a single analog signal that can be fed into existing microscopy hardware for direct visualization, effectively replacing the common PMT analog input of a multiphoton microscope. This enables the seamless and unrestricted use of existing MPM data acquisition hardware and related control, visualization, and analysis software (NI DAQ and ScanImage2023^23^ in our case).

### Selection and Synthesis of an Organic SWIR Dye for Vascular Imaging

In vivo imaging in the SWIR region poses the challenge of adequate biocompatible fluorescent labeling. While several promising nanomaterials do exist,^24−26^ many of them are either toxic or show poor solubility in water. A common choice is the commercially available dye cardiogreen (ICG), which has demonstrated excellent biocompatibility.^27^ However, it only has a relatively short half-life time (~minutes) in the bloodstream and a diminishing absorption cross section in the SWIR region above 1000 μm.^28^ We thus identified the dye LZ-1105^18^ with absorption and emission spectra that overlap well with available SWIR laser sources and our SNSPD array sensitivity (Figure 1). In particular, the absorption spectrum of LZ-1105 shows a prominent shoulder at 900 nm, which suggests efficient two-photon excitation at 1700 nm. In order to efficiently produce LZ-1105 for in vivo imaging applications, we developed our own alternative and robust synthesis pipeline for this dye (see Methods, SI Note 1, and SI Figure 1). While independent verification of the two-photon cross section is generally difficult and outside the scope of this work, we found LZ-1105 to be bright upon 2P excitation with femtosecond-pulsed excitation in vitro and in vivo, with the cross section not varying significantly between 1650 and 1750 nm.

### In Vivo Deep Brain Imaging

To investigate the suitability of the LZ-1105 dye and explore the performance of the SNSPD array for deep tissue microscopy, we performed in vivo mouse brain vascular imaging experiments. For this, we prepared mice with cranial windows over the visual and motor cortex areas and performed imaging experiments with the mouse head-fixed and anesthetized (see Methods). An intravenous injection of the synthesized LZ-1105 dye into the mouse tail vein was performed with roughly 100 μL of dye solution (in saline buffer) at an appropriate concentration, targeting a dye dosage of 5 mg/kg. This dosage was considered safe based on ref (18), and no adverse effects or evidence of toxicity has been observed.

**Figure 4:**
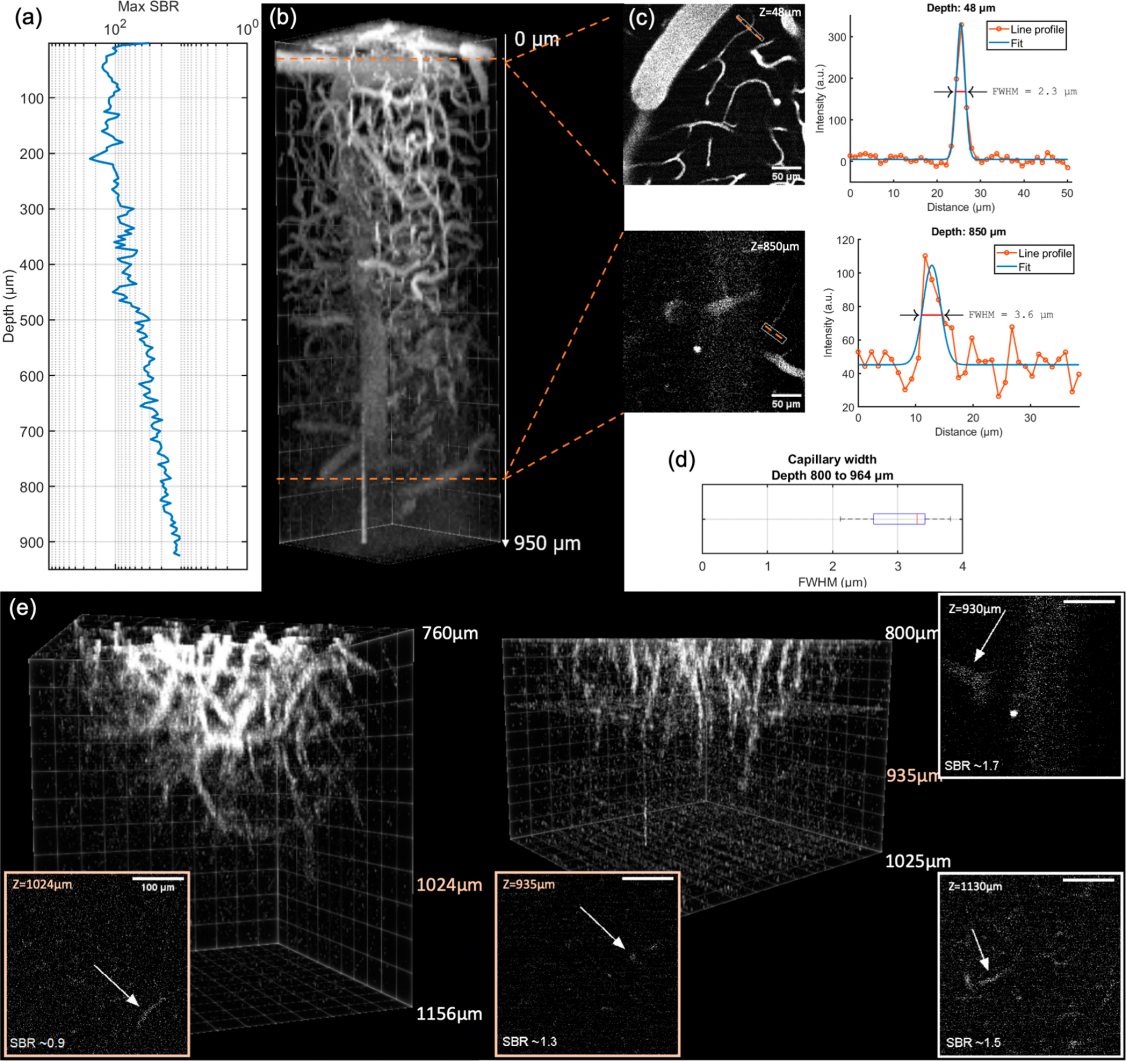
Experimental in vivo deep brain imaging with a custom SNSPD array multiphoton microscope. (a,b) 3D image stack of an adult mouse brain vasculature down to a maximum depth of 950 μm (from the brain surface), and corresponding signal-to-background ratio (SBR) throughout the image volume. The stack consists of 186 slices at 5 μm z-intervals. Each slice is 256 × 256 pixels and covers a ∼300 μm field of view. Dwell time is 6.55 s per z slice (10 frames averaged, 10 laser pulses per pixel). Images recorded in analog summation mode. (c) Exemplary slices at 48 and 850 μm depth showing high SBR and capillary details. Line profiles over blood vessels are shown on the right, with a fit that indicates achievable spatial resolution. (d) Statistical quantification of capillary vessel diameter at large image depth (800–964 μm; *n* = 6), indicating an upper bound of the achievable lateral spatial resolution at this depth. (e) Deep image stacks covering vasculature between 760 and 1156 μm, indicating the highest possible imaging depth at which SBR falls to ∼1. Arrows point to vascular structures. Exemplary data were obtained from four different experiments using three different mice.

For our experiments, we utilized 1700 nm pulsed excitation at a 1 MHz repetition rate and 60 fs pulse duration to efficiently two-photon excite the LZ-1105 dye. Care was taken not to exceed 2 nJ per pulse at the focus and 100 mW average power to prevent optical damage and/or heating of the brain, respectively.^5,10^ With these conditions, we acquired image stacks as shown in Figure 4, where arteries and veins throughout the cortex can clearly be resolved and the images retain high signal-to-background ratios (SBRs) down to a maximum depth of ∼1150 μm, i.e., below the cortex, where SBR reaches ∼1. This represents the depth limit for our SNSPD array-based MPM. At the deepest imaging depth of ∼1.1 mm, the laser power was ∼100 mW with an integration time of approximately 10 s. We note that the achievable imaging depth was predominantly limited by the available concentration of our vascular label and laser power and repetition rate, and that in principle, larger imaging depth could be obtained for sparser and/or higher concentration labeling as well as more optimized pulse energies.

### Experimental Investigation of Depth Limit and Spatial Resolution

To verify the efficient collection of fluorescence throughout the entire imaging depth, we also analyzed the photon count distribution on the SNSPD array. We found that the center pixel of the array on average receives around 8000 counts (photons) per second, which is well below the maximum count rate of 10^6^ photons per second. For single photon counting with a pulsed source at 1 MHz repetition rate and given that this is an average rate over the entire image frame, we estimate that the brightest spots in the sample generate a count rate of ∼80–100k photons per second. In the case of multiphoton excitation deep inside highly scattering tissues in particular, relatively lower repetition rate lasers are necessary (0.5–10 MHz) in order to generate sufficient signal without exceeding safe power limits.^5,29,30^ For efficient detection, it is thus essential to spread the signal over multiple pixels, and the array detector provides the dynamic range that makes this kind of deep imaging experiment possible.

To characterize the achievable spatial resolution, we analyzed the narrowest blood vessels in each image frame, establishing an upper bound estimation for the lateral resolution (Figure 4c). Specifically, two slices were selected, one at a superficial depth of 60 μm and another at 850 μm depth, and line profiles are plotted across the vessel. We also note that this procedure itself is conservative since blood vessels are in general larger (4.2 ± 0.4 μm,^31^) than the lateral extent of the excitation PSF. On average, we found an average vessel width of 3.0 ± 0.6 μm at an average depth of 856 μm (Figure 4d). Therefore, we conclude that our SNSPD detection system does not negatively affect the achievable spatial resolution and that our combination of low-noise-sensitive detectors with multiphoton excitation enables high-resolution imaging at advanced depths in the infrared fluorescence range.

### Gated Digital Counting Improves Image SBR

**Figure 5:**
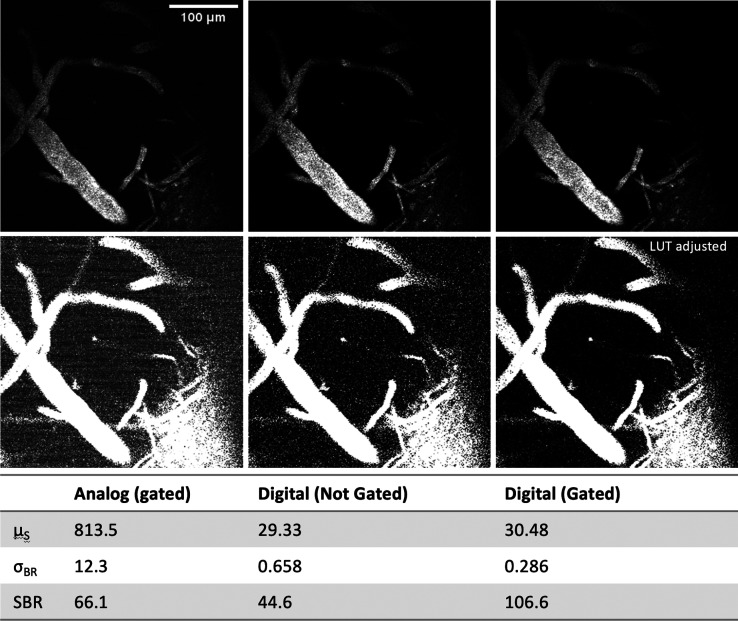
Effect of analogue and digital gating on image contrast. The same image, showing clear signal and background regions, is acquired to compare the achievable SBR between three different modalities. The top row shows the resulting image in which (left) analog summation, (middle) digital counting without and (right) with gating (80 ns width). The bottom row has the contrast adjusted for better visualization of the noise floor. The best SBR is achieved in gated digital mode: μ_S_, mean of signal, σ_BR_, standard deviation of background.

The custom electronics developed for the SNSPD array allow not only an analog summation of the total signal detected by the 24 pixel channels but also digitize them to be used by a FPGA module, which provides counting functionality via a user-defined delay and integration period (see Methods). This allows reconstruction of the image from the binary counting information and adds additional capability to further improve the image SBR by suppressing background noise. We characterized the effects of analog vs counting (digital) mode as well as the role of gating in suppressing background noise. For this, an image at 42 μm depth was acquired with three different modalities, i.e., with analog signal summation and digital counting with and without gating (Figure 5). In the digital mode without gating, all events (photons) are recorded between excitation pulses, leading to the integration of both fluorescence and dark counts or stray light (background noise). The gated image in turn was acquired in counting mode with the gate set 80 ns width following each excitation pulse. As is evident from Figure 5, gated digital counting improves the SBR by ∼60%. This improvement is mainly due to the suppression of background analogue ripple that is caused by RF noise and ground loops but also due to other analogue noise sources (e.g., ADC quantization noise). There is an even greater improvement observed in going from ungated digital to gated digital (∼139%), which is attributed to the effective suppression of dark counts and stray light. Therefore, digital counting mode achieves the best possible SBR at depth.

## Discussion and Conclusions

To summarize, in this work, we developed and characterized a custom multi-element SNSPD array, thereby overcoming a critical technological bottleneck for SWIR bioimaging applications. To the best of our knowledge, this is the first time that a dedicated SNSPD array technology has been developed and integrated into a multiphoton microscope. Together with the synthesis of a near-infrared dye emitting at ∼1100 nm, we established a suitable testbed for proof-of-principle experiments, which clearly shows the potential of this detector technology for deep-tissue microscopy via two-photon excited fluorescence in the SWIR region. In this context, the multi-element SNSPD array provides the following distinct enabling features: Its pixelation provides an increased dynamic range as required by a low-repetition rate, pulsed excitation regime as typically necessary in deep-tissue microscopy. Here, compared to standard PMTs or SiPMs, the SNSPD array provides the low-noise and dark counts necessary for high SBR in low (fluorescence) signal settings, which can further be enhanced by digital gating and postprocessing, as enabled by custom electronics. We nevertheless note that our custom imaging setup including the SNSPD array should be considered a complex, highly optimized research tool and therefore not be directly compared or benchmarked to commercial alternatives (c.f. Figure SI 3, Table SI 1). Although not demonstrated here, we further note that the SNSPD array and its electronics developed in this work could readily be utilized in other challenging bioimaging applications such as fluorescence lifetime microscopy. Furthermore, the fact that the array records the spatial position of the detected photon could be further exploited for image scanning microscopy^32,33^ and related approaches that yield improved spatial resolution or for other methods aimed to improve image contrast by selectively rejecting background or out-of-focus photons in post-processing.

It is noteworthy that here we achieve multiphoton-based imaging depth over 1 mm with an organic dye that is orders of magnitude less bright than quantum dots previously used with SNSPD technology^16,17^ or highly engineered nanoparticles used in 2PM^12,13^ and also without going to lower cross-section three-photon excitation modalities.^4−6^ While these prior works achieved larger overall tissue penetration depth, we highlight that the main motivation of our study was to showcase and demonstrate the potential of a novel, and ultimately scalable, SWIR detector technology. At present, the main limitation of our SNSPD array is its limited overall effective size, which necessitates descanning of the fluorescence and thus only detects near-ballistic light and entails unavoidable and substantial photon loss. Possible further improvements especially with respect to its collection aperture and thus detection efficiency could be achieved by enlarging arrays with more individual pixels,^34^ and/or by utilizing special microlens arrays to enhance the effective fill factor. The current limitation for scaling up the number of pixels is the requirement for driving and reading electronics since a limited number of electrical lines can be sent into the cryostat without exceeding the maximum cooling power. Although it will require substantial improvements, once the overall SNSPD array detection area eventually becomes larger, they could be positioned closer to the objective’s back aperture and hence collect more of the scattered fluorescence light. In light of this, our results with an early prototype and nonoptimized, comparatively inefficient NIR dye are encouraging and will likely spur further work in those areas. With such future optimizations, also on the SWIR probe side and laser repetition rate, we foresee that imaging depth limits can be significantly improved and are likely to surpass current state-of-the-art demonstrations.^12,13^ Higher laser repetition rates would also give access to faster overall imaging speeds, while functionalization of SWIR probes could enable dynamic imaging applications.

Altogether, our work paves the way for more efficient near-IR multiphoton microscopy and may motivate further work on high-gain and low-noise SWIR detectors as well as near-IR fluorescent probes tailored for biological imaging.

## Materials and Methods

### Custom MPM Setup

The core hardware of the 2P near-IR microscope has been described in detail in previous work.^6^ In the following, a brief summary of the instrumentation is provided emphasizing any differences from the details given in ref (6): As a laser source, we also employed a Class 5 Photonics White Dwarf WD-1300-dual laser. The 1700 nm channel used here provided a maximum of over 7 W at a repetition rate of 1 MHz. A motorized half-wave plate followed by a polarizer, in addition to a reflective optical density filter with a static OD = 0.8 attenuation, was used to adapt the power range of the laser, yielding a maximum of 100 mW after the objective. Dispersion precompensation was done by an internal module in the White Dwarf which yielded 60 fs pulses after the objective (Olympus, 25× NA1.05 water immersion). The custom MPM is controlled via ScanImage (VidrioTechnologies) and its associated National Instruments data acquisition system.

The SNSPD array was optically coupled to the microscope by directing the fluorescence into the outermost window of the cryostat (Figure 2a). This is achieved by a long-pass dichroic at 1200 nm (Edmund Optics) situated just before the galvanometric mirror scanning system; therefore, the fluorescence is effectively descanned before it is directed to the SNSPD array. The optical relay to the array is composed of a cage system, protected silver mirrors, and a 1:1 telescope (*f* = 150 mm; Thorlabs achromats, B-Coated). Alignment was optimized by visualizing the size of the detection PSF directly on the array, adjusting it to be centered and for the outer detector elements to have approximately a factor of 10 less counts than the center pixel. This ensures good collection efficiency even in the case of more severe tissue scattering, which results in nonballistic fluorescence and a larger detection footprint on the detector.

### Fabrication of the SNSPD Array

For the deposition of the superconducting NbTiN film, an AJA sputtering machine was used, configured to cosputter Nb and Ti. Contacts, routing, and alignment markers were defined in Au using a laser writer and a standard lift-off process. Subsequently, the meander pattern was patterned using an EBPG and the positive tone AR-P 6200 resist. After patterning, the NbTiN was etched using an SF6 and oxygen plasma. It is important to note that these samples are sensitive to ESD discharge, so long SEM imaging should be avoided and during handling measures should be taken to prevent ESD discharge.

### Characterization of the SNSPD Array

A full characterization of an SNSPD consists of measurement of the quantum efficiency, which states the probability of detecting an incident photon, the dark count rate, and the timing resolution (jitter).

To measure the quantum efficiency of the system, a known number of photons in a given time interval are sent to the system with a linear polarization parallel to the SNSPD meander, which maximizes the detected counts. The details of this type of measurement are explained elsewhere.^35^ By comparing the incident photon rate with the detected photon rate, we experimentally determine the detection efficiency or the quantum efficiency of the system. Note that for this procedure we treat the detector as a bucket detector, i.e., we sum the detected counts for all the pixels while making sure that the incident photons are all arriving on the sensitive area of the array. For determining the dark count rate, we turn off all light sources, cover the SNSPD system, and measure the number of counts detected.

We also characterized the time resolution for single-photon detection using the standard procedure for jitter determination of an SNSPD, as presented in ref (36). Briefly, we use a pulsed laser to synchronize our laser and detect and record a histogram of detected times. The width of this histogram is a measure of the timing jitter for the detector (see Figure 2e).

### Electronic Interface

The SNSPD drivers generate an ∼20 ns long and about 200 mV peak pulse for each detected photon. In order to enable high-quality data processing and to simplify the downstream analysis, we implemented the following pulse processing: The output signal of the driver is cleaned from high-frequency noise by a first low-pass filter. Then, custom-made printed circuit boards (PCBs) standardize the voltage level using a comparator with a user-adjustable threshold and the pulse duration using monoflops, ensuring fixed-length pulses independent of the incoming pulse length. This produces reliable pulses with a 50 ns width and a height of either 3.3 or 5 V (user-selectable) for every photon that hits the SNSPD-array. These signals can be interpreted as digital pulses and are fed directly to the FPGA (Cora Z7–07S) for digital processing. For the analog processing, the 24 channels are scaled down to roughly 300 mV, low-passed, and then summed together to have a single analog channel going into the existing DAQ system of the MPM (NI-5734:4 analog inputs, 80 MS/s 16-bit, absolute maximum voltage ±10 V DC). More details are provided in SI Figure 1.

The electronics discussed above and in Figure 3b not only yield an analogue summation of the total signal detected by the 24-pixel channels of the current SNSPD array but also make all those individual channels available to an FPGA module, which provides counting functionality. For every laser pulse of the excitation laser, the FPGA module resets an on-board counter, which forms the detection time-base. This counter is incremented at the FPGA clock rate of 125 MHz to yield time bins of 8 ns each. A user-defined delay and integration period then form the parameters for an integration gate during which the FPGA will have the channels armed to record a photon arrival. Within the gate, up to one photon can be recorded (an extremely long digital pulse can be optionally recorded as two photons), and outside the gate, nothing is recorded. This yields an array of logical 1 and 0 associated with each pixel and each laser pulse, indicating the arrival of a photon during the integration period associated with that pulse. Some auxiliary pulses (ScanImage’s frame and line triggers) are also recorded by the FPGA. This data is streamed via a TCP/IP server to a binary file along with the aforementioned external reference signals, allowing reconstruction of the image from the binary counting information. A rough image reconstruction is done using the auxiliary synchronization lines, and then by cross-correlating each digital image line with the corresponding analog image line, the remaining synchronization and pixel alignment is achieved.

### Dye Synthesis and Characterization

The synthesis of LZ-1105 was achieved in three steps starting from 2-phenyl-1*H*-indole using a modified protocol of the literature-known procedure^18^ to obtain 4-(2-phenyl-1*H*-indol-1-yl)butane-1-sulfonate (**1**) in the first step after alkylation with 1,2-oxathiane 2,2-dioxide. The key for the synthesis of LZ-1105 was the stepwise reaction of compound **1** with acetyl chloride in the presence of acetic anhydride in toluene to afford dimeric compound **2** in very good yield. The following reaction with readily available *N*-((*E*)-(2-chloro-3-((*E*)-(phenylimino)methyl)cyclopent-2-en-1-ylidene)methyl)anilinium chloride (**3**)^37^ in methanol in the presence of hydrochloric acid provided the target dye LZ-1105. Further details on the synthesis procedure can be found in the Supporting Information (SI Note 1 and SI Figure 1).

### Animal Preparations and Imaging Procedure

This work followed the European Communities Council Directive (2010/63/EU) to minimize animal pain and discomfort. All procedures described in this paper were approved by EMBL’s committee for animal welfare and institutional animal care and use, under protocol numbers RP170001 and 22–004_HD_RP. Experiments were performed on male and female, 7–24-week-old C57Bl6/j or homozygous Thy1–EGFP-M (Jax no. 007788) transgenic mice from the EMBL Heidelberg core colonies. During the course of the study, mice were housed in groups of 1–5 in Makrolon type 2L or 3H cages, in ventilated racks at room temperature and 50% humidity while kept in a 12/12 h light/dark cycle. Food and water were available ad libitum.

The cranial window implantation surgery was performed as follows. 7–8-week-old mice were anesthetized by i.p. injection of a mixture of 40 μL of fentanyl (0.1 mg/mL; Janssen), 160 μL of midazolam (5 mg/mL; Hameln), and 60 μL of medetomidin (1 mg/mL; Pfizer), dosed in 5 μL/g body weight. Hair over the scalp was removed with hair removal cream, and eye ointment was applied (Bepanthen, Bayer). Anesthetized mice were then subcutaneously injected with 1% xylocain (AstraZeneca) under the scalp as preincisional local anesthesia and placed in a stereotaxic frame (RWD Life Science, model 68803). The skin and periosteum over the dorsal cranium were removed with fine forceps and scissors to expose the bone. A 4 mm diameter circular craniectomy was made centered over the right visual cortex using a dental drill (Microtorque, Harvard Apparatus, 2.5 mm posterior and 2.5 mm lateral to Bregma). Close care was taken to preserve the integrity of the dura and to avoid bleeding. A round 4 mm coverslip (around 170 μm-thick, disinfected with 70% ethanol) was placed over the craniectomy, with a drop of saline between the glass and the dura. The craniectomy and cranial window were sealed using acrylic dental cement (Hager Werken Cyano Fast and Paladur acrylic powder), and a customized metal headbar was cemented to the skull for head fixation under the microscope. The skin wound around the surgical area was also closed with a dental acrylic. After surgery, mice received pain relief (Metacam, Boehringer Ingelheim, subcutaneous injection, 0.1 mg mL–1, dosed 10 μL g–1 body weight), and anesthesia was antagonized by subcutaneous injection of a mixture of 120 μL of sterile saline, 800 μL of flumazenil (0.1 mg/mL; Fresenius Kabi), and 60 μL of atipamezole (5 mg/mL; Pfizer) dosed in 10 μL/g body weight. Mice were single-housed after cranial window implantation and had a recovery period of at least 3 weeks before further experiments to resolve the inflammation associated with this surgery.^38^

For imaging, a chronic window implanted Thy1-EGFP-M or WT mouse was head-fixed under the microscope and anaesthetized with isoflurane vapor mixed with O2 (5% for induction and 1–1.5% for maintenance). An intravenous injection into the mouse tail vein was performed with roughly 100 μL of dye solution (in saline buffer) at an appropriate concentration targeting a dye dosage of 5 mg/kg. This dosage was considered safe based on ref (18), and we did not observe evidence of toxicity even after three separate injections at this dosage. Care was taken not to exceed 2 nJ per pulse at the focus and average power below 100 mW to prevent photo damage to the brain. With these conditions, the imaging stacks in Figure 4 were acquired. All reported depth values were recorded by the microscope translation stage, which was referenced (“zeroed”) at the pia, which was regarded as the surface of the brain. Any larger movements or experimental interruptions were followed by a depth check at the surface to ensure lack of drift, with the cranial window being rigorously leveled at the beginning of the experiment.

For image analysis and 3D visualization, Fiji^39^ and ClearVolume^40^ was used, respectively. For the SBR calculation, blood vessels were segmented using the software package.^41^ One frame is minimally hand annotated to train the model to recognize blood vessels, resulting in probability maps for both the signal and background for the entire stack. These probability maps are thresholded conservatively (high probability) to generate binary signals and background masks. These masks are then fed into the FIJI ROI editor as ROIs, and measurements defined on them, in the case of the signal, mean, and maximum, and in the case of the background, mean, and standard deviation. Mean and max SBR figures are then generated by subtracting the background mean from the signal mean and max, respectively, and divided by the background standard deviation. For spatial resolution analysis, 5- or 10-pixel wide line profiles were plotted across blood vessels. The width of the line determines how many pixels are averaged for each point in the profile. We note that this averaging procedure can only result in a wider profile and that a blood vessel cannot be observed to be narrower than the excitation PSF. Therefore, the calculated resolution is indeed a conservative, upper bound on the PSF size.

All raw imaging data including full stacks that are shown in Figure 4 and 5 are available on Zenodo at: https://zenodo.org/doi/10.5281/zenodo.10926482.

## Supporting Information Available

Additional experimental details, materials, and methods, including photographs of experimental setup (PDF); in particular, (1) chemical synthesis and characterization of LZ-1105; (2) additional details on the custom electronic interface; (3) performance comparison of current detector technologies (PDF)
