# Supplementary material for: Deep Mouse Brain Two-Photon Near-Infrared Fluorescence Imaging Using a Superconducting Nanowire Single-Photon Detector Array
Source: ACS Photonics. 2024 Sep 11;11(10):3960–71. doi: 10.1021/acsphotonics.4c00111 (PMC11487655; doi:10.1021/acsphotonics.4c00111)
Supplement: Supplementary file 1 — ph4c00111_si_001.pdf [file ph4c00111_si_001.pdf]

Supplementary Information

Deep mouse brain two-photon near-infrared fluorescence imaging using a superconducting nanowire single-photon detector array

Amr Tamimi[1], Martin Caldarola[2], Sebastian Hambura[1], Juan C. Boffi[1], Niels Noordzij[2], Johannes W. N. Los[2], Antonio Guardiani[2], Hugo Kooiman[2], Ling Wang[1], Christian Kieser[1], Florian Braun[3], Mario A. Usuga Castaneda[2], Andreas Fognini[2], Robert Prevedel[1,4-7]

[1] Cell Biology and Biophysics Unit, European Molecular Biology Laboratory, Heidelberg, Germany.
[2] Single Quantum B.V, Delft, The Netherlands.
[3] Chemical Synthesis Core Facility, European Molecular Biology Laboratory, Heidelberg, Germany.
[4] Developmental Biology Unit, European Molecular Biology Laboratory, Heidelberg, Germany.
[5] Epigenetics and Neurobiology Unit, European Molecular Biology Laboratory, Rome, Italy.
[6] German Center for Lung Research (DZL), Heidelberg, Germany.
[7] Interdisciplinary Center of Neurosciences, Heidelberg University, Heidelberg, Germany.

Correspondence: M.C. (m.caldarola@singlequantum.com) or R.P. (prevedel@embl.de).

Supplementary Note 1: Chemical synthesis and characterization of LZ-1105

Scheme 1: Synthesis of **LZ-1105**.

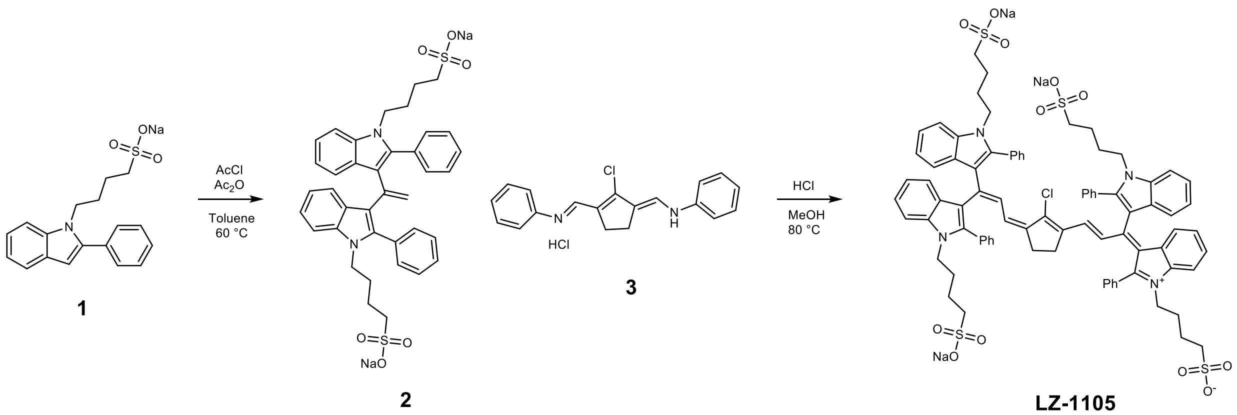

General methods

Reagents were purchased from Sigma Aldrich (Germany) and TCI (Belgium) and used without further purification. All solvents, including anhydrous solvents, were used as obtained from the commercial sources. Air and water-sensitive reagents and reactions were generally handled under argon atmosphere. The reaction progress was monitored by TLC on Merck silica gel plates 60 F254. Detection was executed with a UV-Kabinett HP-UVIS (biostep) at 254 nm or with potassium permanganate staining. Flash chromatographic purification was performed on a Biotage® Isolera One purification system using Biotage® SFär C18 D flash cartridges. Nuclear magnetic resonance spectra were recorded on a Bruker Avance (400 MHz) NMR System at 298 K. Chemical shifts (δ) are given in parts per million (ppm), coupling constants (J) given in Hertz (Hz) and multiplicity is reported using standard abbreviations. UHPLC/MS analyses were performed on Agilent 1290 series equipment consisting of an Agilent 1290 quaternary pump, a 1290 sampler, a 1290 thermostated column compartment and a 1290 Diode array

detector VL+ equipped with a quadrupole LC/MS 6120 and an Infinity 1260 ELSD. The analytical column used was a Titan C18 UHPLC Column (2.1 X 30 mm, 1.9 µm) operated at 40 °C and 1.5 ml/min flow rate with a gradient (10% to 15% B in 0.4 min, 15% to 100% B in 1.6 min, 100% B for 0.5 min) using water (A) and acetonitrile (B), both containing 0.1% TFA, as solvents. Compound purity was determined by ELSD monitoring. *N*-((*E*)-(2-chloro-3-((*E*)-(phenylimino)methyl)cyclopent-2-en-1-ylidene)methyl)anilinium chloride **(3)** was synthesized following the literature procedure [31].

Synthesis

Sodium 4-(2-phenyl-1H-indol-1-yl)butane-1-sulfonate (1).

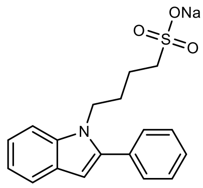

Compound **1** was synthesized according to literature known procedure [15]. In brief: To 2-phenyl-1H-indole (1.93 g, 10.0 mmol) in anhydrous tetrahydrofuran (50 mL) 60% sodium hydride (252 mg, 10.5 mmol) was added at 0 °C and the mixture was stirred at 0 °C for 1 h. 1,2-Oxathiane 2,2-dioxide (1.36 g, 10.0 mmol) was added and the reaction mixture was stirred at 100 °C for 2 h. After cooling to room temperature, the mixture was concentrated under reduced pressure and treated with isopropanol. The resulting white solid was collected by filtration and dried in vacuo to obtain 3.51 g (8.68 mmol, 87%) of the analytically pure title compound. ^{1}H NMR (400 MHz, DMSO-*d*$_6$) δ 7.58 – 7 49 (m, 6H), 7.48 – 7.41 (m, 1H), 7.16 (t, *J* = 7.7 Hz, 1H), 7.05 (t, *J* = 7.1 Hz, 1H), 6.50 (s, 1H), 4.17 (t, *J* = 7.6 Hz, 2H), 2.29 (t, *J* = 7.6 Hz, 2H), 1.71 – 1.58 (m, 2H), 1.50 – 1.36 (m, 2H).

Compound LZ-1105.

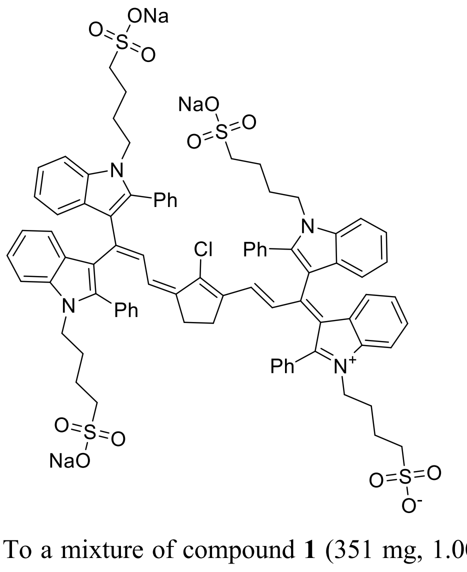

To a mixture of compound **1** (351 mg, 1.00 mmol) and acetic anhydride (0.24 mL, 2.50 mmol) in toluene (1.0 mL), acetyl chloride (71 µL, 1.00 mmol) was added and the reaction mixture was stirred at 60 °C for 4 h. The resulting solid was separated and used in the next step without further purification. ^{1}H NMR (400 MHz, $CDCl_3$) δ 7.45 (d, *J* = 8.2 Hz, 2H), 7.29 – 7.20 (m, 6H), 7.29 – 7.12 (m, 4H), 7.12 – 7.05 (m, 6H), 6.91 (t, *J* = 7.5 Hz, 2H), 3.93 – 3.83 (t, *J* = 7.1 Hz, 4H), 2.35 (t, *J* = 7.5 Hz, 4H),1.52 – 1.42 (m, 4H), 1.42 – 1.33 (m, 4H), MS (ESI): 683

(M + H)$^+$.To the residue and compound **3** (78.0 mg, 0.23 mmol) in Methanol (0.5 mL), 1.25 M HCl in Methanol (0.5 mL) was added and the reaction mixture was stirred at 80 °C for 2 h. The mixture was evaporated and the crude material was directly purified on a Biotage® SFär C18 D – 12 g column using a water/ methanol gradient (both containing 0.1% TFA) to obtain 46 mg (13%) of the title compound. ^{1}H NMR (400 MHz, DMSO-d_6) δ 7.70 (d, J = 8.3 Hz, 4H), 7.55 – 7.16 (m, 26H), 7.11 – 7.01 (m 4H), 6.80 (d, J = 13.3 Hz, 4H), 6.11(bs, 2H), 4.12 (bs, 8H), 2.39 (bs, 8H), 1.79 (bs, 4H), 1.66 (bs, 8H), 1.44 (bs, 8H), UHPLC: t_R = 2.11 min, MS (ESI): 1488 (M + H)$^+$.

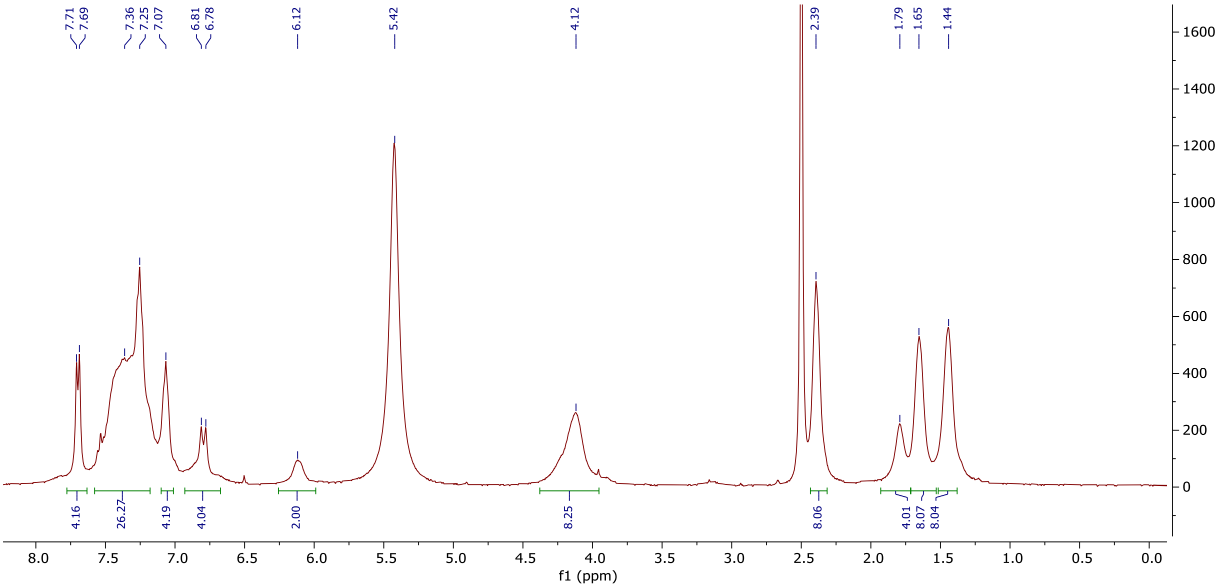

Figure SI 1: ^{1}H NMR (400 MHz, DMSO-d_6) spectrum of **LZ-1105**.

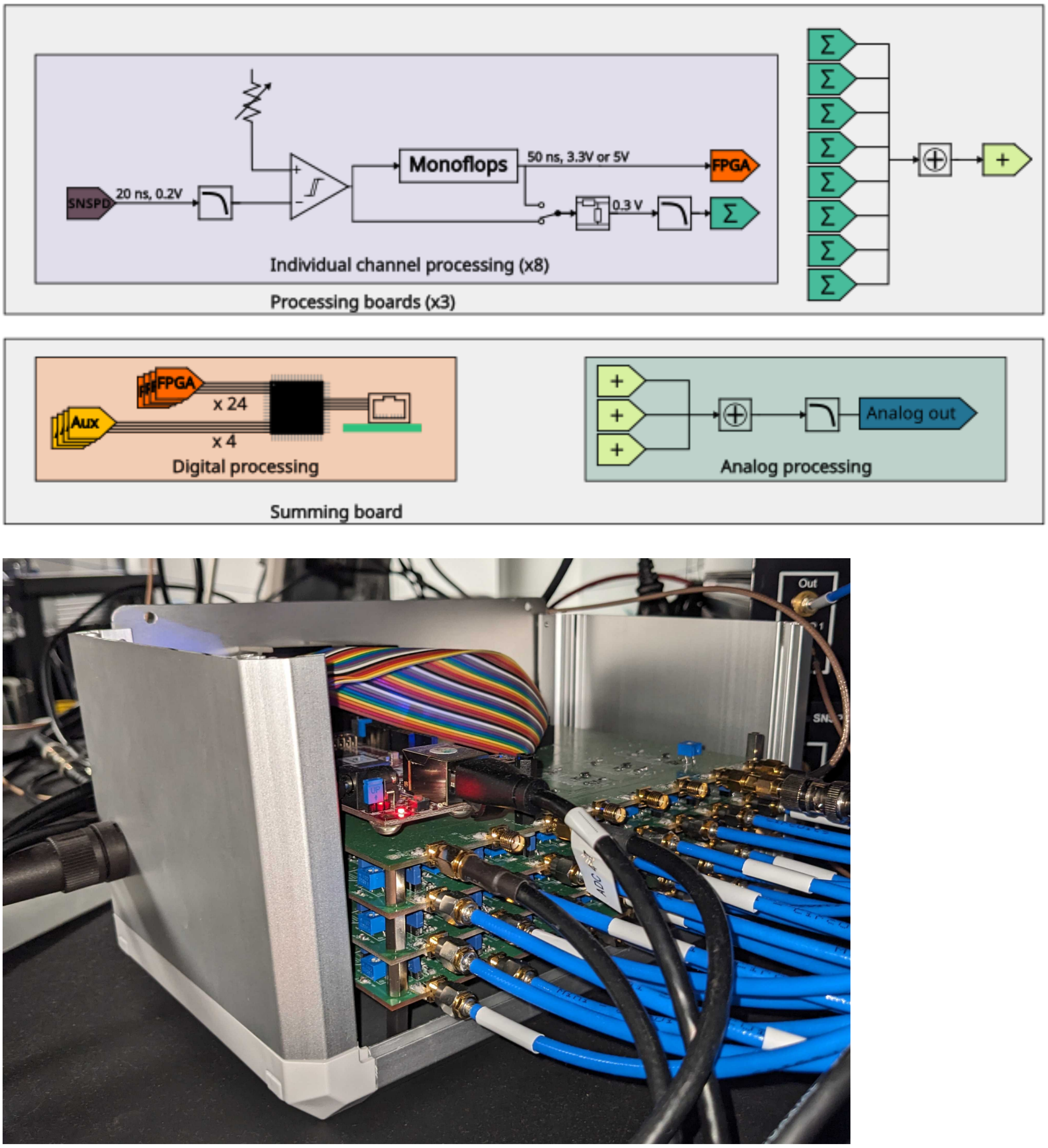

Figure SI 2: Custom electronic interface. (Top) The 8-channel signal processing board. 20 ns and 200 mV pulses are converted into one 50 ns and 300 mV pulse (for analog processing) and one 50 ns digital pulse (3.3 V or 5 V logic high) and the 8 analog signals are summed. Three separate boards are combined to allow recording of 24 independent channels. (Middle) A dedicated PCB board sums the analog signal of the 3 previous boards into a single analog output. It also serves as an intermediate between the digital outputs and the FPGA board, allowing only one connector to be used between the FPGA and this setup. The auxiliary signals such as laser triggers are also sampled here. (Bottom) A picture of the physical device where the bottom 3 boards are the processing boards, the summing board directly above, and the FPGA on top.

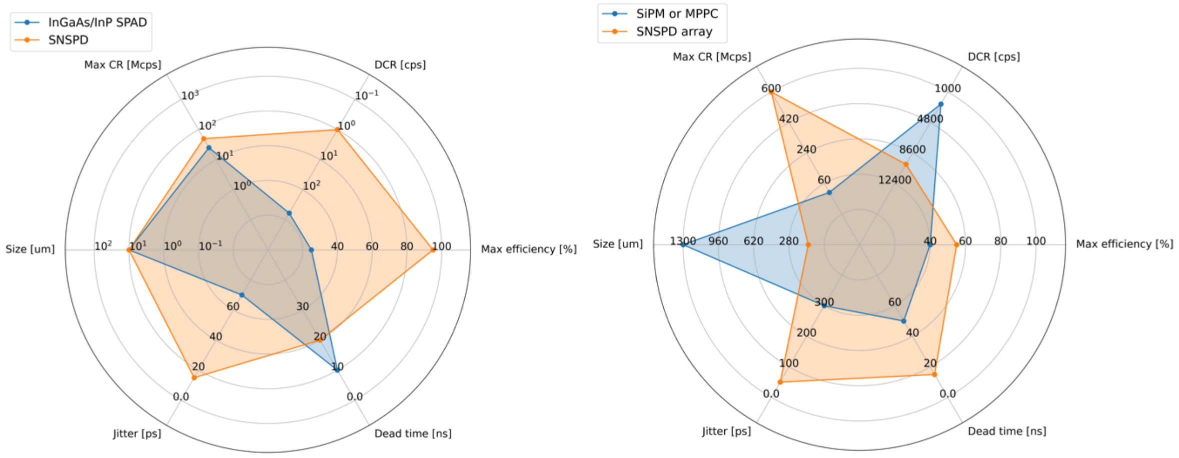

Figure SI 3: Performance comparison of current detector technologies. (Left) Plot comparing the performance of single-pixel SWIR SPAD and an SNSPD optimised for 1550 nm. (Right) Plot comparing the performance of a SiPM with an array of SNSPDs optimised for 1550 nm. Note that the wavelength range for SiPM/MPPCs is significantly different since silicon is only sensitive to visible light. The most relevant parameters for deep 2PE imaging are: size, quantum efficiency and dark count rate (DCR). We further note that our SNSPD array based imaging system should be considered a highly-optimized, complex research tool and should therefore not be directly compared or benchmarked to commercial instruments. Note that some values not reported in the datasheet were estimated.

Table SI 1: Performance comparison of current detector technologies.

	Si SPAD	Si SPAD array	InGaAs/InP SPAD	VIS PMT (photon counting head)	SWIR PMT	SiPM or MPPC	SNSPD	SNSPD array
**Manufacturer**	MPD	Pi imaging	MPD	Hamamatsu	Hamamatsu	Hamamatsu	Single Quantum	Single Quantum
**Exemplary Model**	PD-020-CTE	SPAD23	PI-010-SM	H11123	H10330C-75	S13366-1350GD	EOS - Excellence	SQCam
**Peak detection efficiency [%]**	48	55	25	~21	2	40	95	55
**Peak efficiency wavelength [nm]**	550	520	1200	420	1200	450	1550	1550
**Bandwidth [nm][i]**	250	300	500	250	700	300	150	150
**DCR [cps]**	25	100	600 to 1500	100 to 200	2.5e5	2.5e3 to 7e3	<1	<1e4
**After- pulsing probability [%]**	0.1 to 3	0.1	2			0.1	0	0
**Maximum detection rate [Mcps][ii]**		7.8 (per pixel)		5		8	50	600
**Diagonal sensitive area [um]**	20	150	10	2500	1600	1300	10	94
**Number of pixels**	1	23	1	1	1	667	1	36
**Detector fill factor[%]**	100		100	100	100		100	80
**Pixel pitch [um]**	NA	20	NA	NA	NA	50	NA	11
**Jitter [ps]**	35	85 @ 510 nm	70		367		15	50
**Dead time [ns][iii]**	77	50					20	15

[i] Considered as the FWHM around the main peak
[ii] Considered as the count rate at which the efficiency is half the peak efficiency (3 dB point).
[iii] Dead time per pixel.

Table SI 1: Performance comparison of current detector technologies. This table lists the current performance of various detectors technologies in both the visible as well as SWIR regime. Note that some values not reported in the datasheet were estimated.
